# When a left atrial mass is not a thrombus: pulmonary venous tumour extension in lung cancer

**DOI:** 10.1093/ehjcr/ytag757

**Published:** 2026-10-14

**Authors:** Resha Reya Ganthan, Francesco Rotatori

**Affiliations:** Department of Internal Medicine, Richmond University Medical Center, 355 Bard Avenue, Staten Island, NY, USA; Department of Cardiology, Richmond University Medical Center, 355 Bard Avenue, Staten Island, NY 10310, USA

**Keywords:** Lung adenocarcinoma, Cardiac metastasis, Left atrial mass, Pulmonary vein invasion, Multimodality imaging, Tumour embolization

## Summary

Direct extension of lung malignancy into the left atrium through the pulmonary veins is an uncommon manifestation of advanced disease that may mimic intracardiac thrombus. This case highlights the value of multimodality imaging in distinguishing these entities and informing management.

## Case description

A 58-year-old man with Stage IV lung adenocarcinoma, chronic respiratory failure with ventilator dependence, and prior pulmonary embolism presented with acute respiratory distress. Computed tomography (CT) demonstrated a large left hilar mass, pleural effusion, mediastinal lymphadenopathy, and a 3.8 × 3.6 cm left atrial filling defect initially concerning for thrombus, prompting systemic anticoagulation with heparin.

Contrast-enhanced transthoracic echocardiography demonstrated a 3.6 × 2.7 cm mass arising from the posterior left atrial wall with internal contrast enhancement, supporting vascularity and favouring tumour over bland thrombus^1^. CT demonstrated continuity of soft tissue from the left hilar malignancy through the pulmonary venous system into the left atrium, strongly supporting direct tumour extension^2,3^ (*Figure 1*). The diagnosis was based on multimodality imaging without histologic confirmation; superimposed thrombus could not be excluded.

The clinical course was complicated by multifocal ischaemic strokes, potentially resulting from embolization of friable tumour material and/or superimposed thrombus. Anticoagulation was continued given the prior pulmonary embolism, malignancy-associated thrombotic risk, and possibility of superimposed thrombosis, although its benefit for tumour-related embolization remains uncertain. Given disseminated malignancy, poor functional status, and lack of candidacy for surgical or oncologic intervention, a palliative approach was pursued.

**Figure 1:**
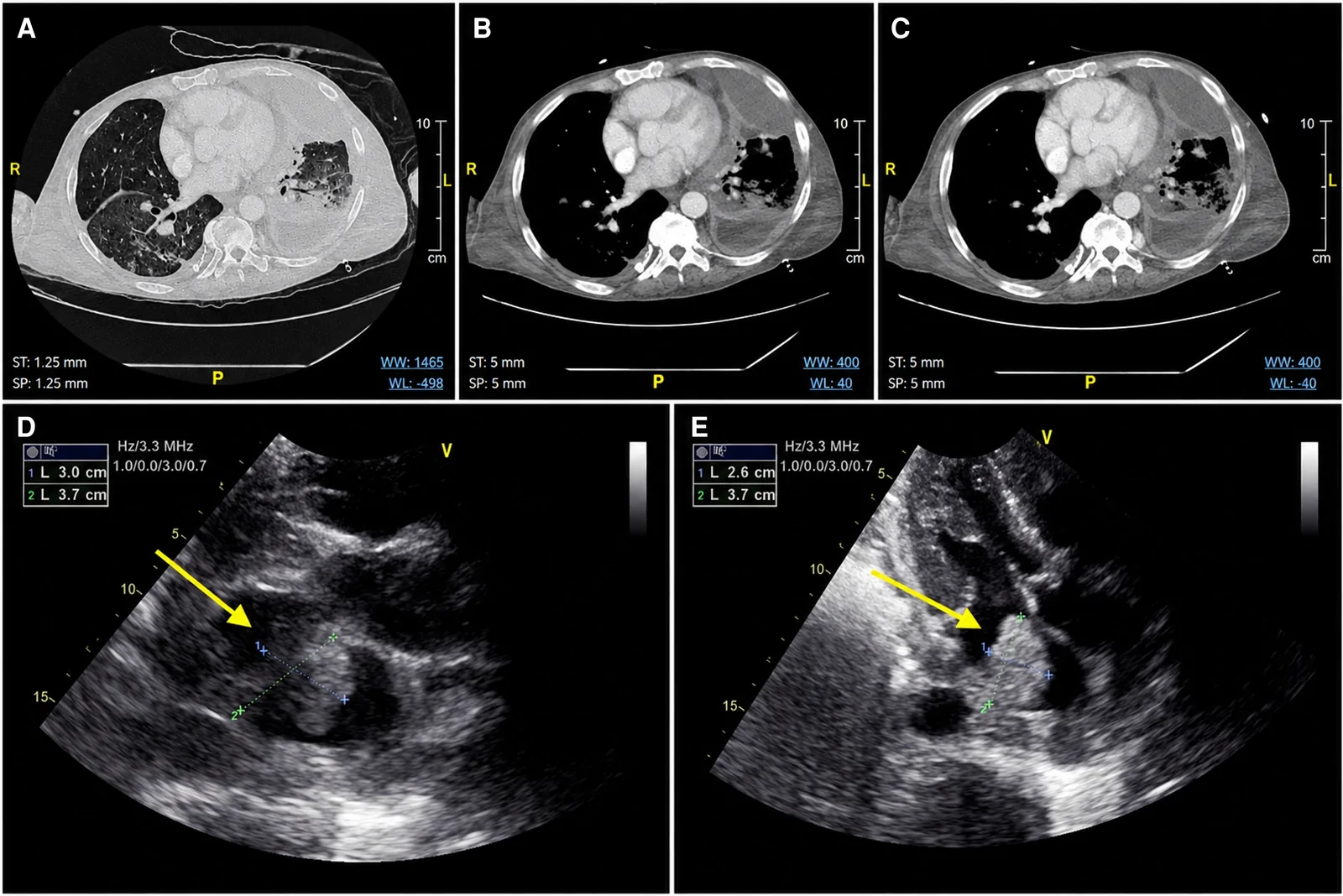
Multimodality imaging demonstrating pulmonary venous tumour extension into the left atrium. (*A*) Axial chest computed tomography (CT) demonstrating the large left hilar malignancy. (*B–C*) Consecutive contrast-enhanced CT images demonstrating continuity of soft tissue between the left hilar malignancy, pulmonary venous system, and left atrium, supporting direct pulmonary venous tumour extension. (*D*) Transthoracic echocardiography demonstrating a large mass arising from the posterior left atrial wall (arrow). (*E*) Contrast-enhanced transthoracic echocardiography demonstrating internal enhancement of the left atrial mass (arrow), supporting vascularity and favouring tumour over bland thrombus.

## Data Availability

All data relevant to the study are included in the article.

## Notes

**Consent:** Written informed consent for publication was obtained.
